# A cross-sectional study of demographic, environmental and parental barriers to active school travel among children in the United States

**DOI:** 10.1186/1479-5868-11-61

**Published:** 2014-05-09

**Authors:** Palma Chillón, Derek Hales, Amber Vaughn, Ziya Gizlice, Andy Ni, Dianne S Ward

**Affiliations:** 1 Department of Physical Education and Sport, School of Sport Sciences, University of Granada, Ctra. Alfacar, s/n, Granada 18011, Spain; 2 Department of Nutrition in the Gillings School of Global Public Health, Chapel Hill, NC 27599, USA; 3 Center for Health Promotion and Disease Prevention, University of North Carolina at Chapel Hill, 1700 Martin L. King Jr. Blvd, CB 7426, Chapel Hill, NC 27599, USA; 4 Department of Biostatistics, University of North Carolina at Chapel Hill, 122 1501 Baity Hill, CB 7420, Chapel Hill, NC 27599, USA

**Keywords:** Correlates, Active travel, Barriers, Children, Walk to school

## Abstract

**Background:**

Promoting daily routine physical activities, such as active travel to school, may have important health implications. Practitioners and policy makers must understand the variety of factors that influence whether or not a child uses active school travel. Several reviews have identified both inhibitors and promoters of active school travel, but few studies have combined these putative characteristics in one analysis. The purpose of this study is to examine associations between elementary school children’s active school travel and variables hypothesized as correlates (demographics, physical environment, perceived barriers and norms).

**Methods:**

The current project uses the dataset from the National Evaluation of Walk to School (WTS) Project, which includes data from 4^th^ and 5^th^ grade children and their parents from 18 schools across the US. Measures included monthly child report of mode of school travel during the previous week (n = 10,809) and perceived barriers and social norms around active school travel by parents (n = 1,007) and children (n = 1,219). Generalized linear mixed models (GLMM) with log-link functions were used to assess bivariate and multivariate associations between hypothesized correlates and frequency of active school travel, assuming random school effect and controlling for the distance to school.

**Results:**

The final model showed that the most relevant significant predictors of active school travel were parent’s perceived barriers, specifically child resistance (Estimate = −0.438, p < 0.0001) and safety and weather (Estimate = −0.0245, p < 0.001), as well as the school’s percentage of Hispanic students (Estimate = 0.0059, p < 0.001), after adjusting for distance and including time within school cluster as a random effect.

**Conclusions:**

Parental concerns may be impacting children’s use of active school travel, and therefore, future interventions to promote active school travel should more actively engage parents and address these concerns. Programs like the Walk to School program, which are organized by the schools and can engage community resources such as public safety officials, could help overcome many of these perceived barriers to active transport.

## Background

Promoting daily routine physical activities, such as active travel to school, may have important health implications. Studies conducted across multiple countries have consistently shown that children who walk or bike to school have higher daily levels of physical activity (PA) than those who travel to school by car or bus [1,2]. Some studies have even shown active commuting to school to be associated with better weight and metabolic outcomes [3,4], and biking to school to be associated with higher cardiorespiratory fitness [5,6]; however, results across studies have been less consistent [7]. While active commuting appears to have important health benefits, the proportion of students who walk or bike to school in the US has declined substantially over the past 40 years [8,9]. While 41% of students walked or biked to school in 1969, this proportion decreased to 10% in 2009. In order to reverse this trend, practitioners and policy makers must understand the variety of factors that influence whether or not a child uses active school travel.

Several literature reviews [2,10-12] have highlighted factors associated with children’s use of active school travel, including socio-demographic characteristics (e.g., ethnicity, income), attributes of the physical environment (e.g., distance, sidewalk width, weather), and perceptions of the social environment (e.g., social norms). These results generally suggest that children from non-white ethnic backgrounds and/or low-income families, and those who live close to school and travel with friends are more likely to engage in active school travel than their counterparts. While these reviews are helpful in identifying both inhibitors and promoters of active school travel, no study has combined these putative characteristics in one analysis. The methodological limitation of focusing only on the significance of association between a single variable and active school travel has been previously reported [13]. Understanding the main correlates of active school travel may help practitioners and policy makers design and implement more effective active transportation intervention programs.

The study uses data from the National Evaluation of Walk to School project [14,15]. Data from this project provide the opportunity to explore the interplay between socio-demographic characteristics, the physical environment, and the social environment – factors that have been highlighted in a recent review as important correlates of active school travel [16]. More specifically, this paper will use data from a large and geographically dispersed sample of children and parents to describe both individual and combined associations between use of active school travel and several hypothesized correlates including socio-demographic factors, physical factors, and parent and child perceptions).

## Methods

### Study design and sample

Data were collected as part of the National Evaluation of Walk to School Project, a nationwide study of the Walk to School (WTS) program and its impact in the US. All procedures for this study were approved by the Institutional Review Board at the University of North Carolina at Chapel Hill. The first phase of this project involved a national survey of WTS program coordinators [15], from which 14 schools were randomly selected to participate in a more intensive evaluation (phase 2). These 14 “case” schools represented five geographic regions (Alaska and Northwest (NW), California (CA), Southcentral (SC), Southeast (SE), and Northeast (NE)), as well as three levels of WTS program implementation (low, medium, high) [14,15]. In addition, a “control” school (with no previous participation in WTS) was also identified from each of the five regions and recruited to participate for a total sample of 19 schools. Data from one school were excluded from the current analysis because the school was located in a rural area where active transportation to the school was not possible (with WTS efforts being used to promote activity through a “walk at school” effort). Thus, data from 18 elementary schools in nine states were available.

### Recruitment and data collection procedures

Recruitment was a two-step process that first required recruiting a sample of schools, then recruiting a sample of families at those schools with a child in 4^th^ or 5^th^ grade. School principals were the target for initial invitations, which were sent along with a fact sheet describing the nature and purpose of the study. When necessary, a complete description of study protocols was submitted to the local school board for approval. Schools that agreed to participate signed a memorandum of understanding and designated a member of the school staff to serve as the local coordinator for the project.

Once a school agreed to participate, an onsite visit was conducted during the fall of 2003. During this visit, project staff conducted an assessment of the school’s active travel environment. In addition, project staff trained the local coordinator on recruitment and data collection procedures so that they could assist with distribution of materials supplied by the project (e.g., recruitment packets and surveys) and return on completed surveys to the project office.

Recruitment of families was initiated after this visit. Information packets were sent home to all eligible families. Packets included a project fact sheet, a request for family participation, and a parent school travel and safety survey. Given the minimal risk of this study and lack of any identifying information, return of the parent survey was considered as consent for their participation. IRB also approved the use of passive consent for children, where consent forms were returned only if parent did not want child to participate.

Approximately two weeks after distributing these parent information packets, child surveys began to be administered. Child surveys were completed during class at regularly time points throughout the school year (from October 2003 to May 2004). Surveys included a *travel recall* that was collected monthly and a *school travel and safety survey* (similar to the parent survey) that was collected once in the fall and again in the spring.

Toward the end of the school year, onsite visits were repeated to assess any changes in the school’s active travel environment. Additionally, a second round of parent school travel and safety surveys was also distributed.

### Measures

#### Active school travel

Use of active school transportation was assessed using a travel recall instrument with acceptable reliability and validity evidence [17]. Travel recalls asked children to report how often they used various modes of travel to get *to* and *from* school during the previous week, including: walking, riding a bike, riding a car or riding a bus. For example, questions were phrased: “Last week, how many days did you *walk* to school?” Trips to and from school were asked separately, resulting in a total of 8 questions. Students could answer 0 to 5 for each question. The main outcome used in the statistical analysis was total number of active trips per week. This included trips to and from school made by walking or biking, scores ranged between 0 and 10. The travel recalls were also used to identify the percentage of “active” travelers (children with ≥ 4 active travel trips per week) at each school.

#### School travel and safety

School travel and safety surveys were completed by both parents and children. The parent version of the survey assessed perceived barriers and the social norms around active school travel. Given the known influence of distance on use of active school travel, the survey began by asking parents to estimate the distance between home and school (response options: less than ½ mile, between ½ and 1 mile, between 1 and 1 ½ miles and more than 1 ½ miles). From this information, we calculated the percentage of families at a given school living within 1 mile radius. Parents were then asked to identify reasons why they “cannot or do not allow their child to walk or bike to school” from a checklist of 22 barriers commonly reported in the literature and identified from formative work [18]. Using exploratory factor analysis we identified five barrier factors using 20 of the original 22 items. They included external safety and weather (6 items: bullies, kidnapping, arriving safely to school, weather, unleashed dogs and traffic congestion), suitability of the route (6 items: lack of sidewalks and crosswalks, steep hills, areas without people around, speed and traffic and insufficient daylight in the morning), time issues (3 items: lack of time in the morning and afternoon, more convenient to drop-off or pick-up), no walking companion (3 items: no other kids or adults to walk with, conflicts with work schedule), and child resistance (2 items: child too tired and child does not want walk or bike). Details of this exploratory factor analysis are available as an Additional file 1. Each barrier item was scored 0 (no) or 1 (yes) and an average score was computed for each barrier factor. For ease of use, scores were multiplied by 100 so that they represented the proportion of items within a given barrier factor selected by parents. Higher scores indicate a greater number of perceived barriers to active school travel. School level barrier scores were then computed by averaging scores for a given factor across all parents at the school. Parents’ perceived active school travel norms were assessed with one question, “How often do other people in the neighborhood walk or bike with children to/from school?” (Response options: everyday, a few times a week, a few times a month, a few times a year, or never). The percentage of parents at a given school who responded “everyday” or “a few times a week” was then calculated.

The child version of school travel and safety survey asked students to identify “things that made it hard to walk or bike to school” using a checklist of 13 items. Based on expert opinion, response frequency and previous research, an index was computed for the five most common barriers: traffic, weather, no time in the morning, too tired in the morning and parents don’t let them. A barrier index score was computed for each child by averaging responses to these five barrier items (1 = marked, 0 = not marked). This score was then multiplied by 100 in order to calculate the percentage of these five barriers selected. Higher scores indicated a greater number of perceived barriers to active school travel. School level barrier scores were then computed by averaging scores across all children at the school. The child perceived active school travel norms were estimated by asking “if other kids in their neighborhood walk or bike to/from school”. The outcome was calculated as percentage of children responding “yes”.

#### Physical environment

Two aspects of the physical environment were measured, walkability-bikeability and temperature. The Walkability and Bikeability Suitability Assessment (WABSA) protocol [19] was used to assess the school zone and surrounding area. The walking suitability component captures variables like vehicle traffic and speed, pedestrian signals and suitability of sidewalks, while the bicycle suitability component captures variables like vehicle traffic and speed and suitability of roads (e.g., bike lane presence, width, etc.). The most popular routes were identified by school administrators, and assessment forms were completed on a sample of road segments within a 1-mile buffer of the school by research staff who visited the school on two occasions. Walkability and bikeability scores were continuous values with higher scores indicating a more hazardous environment. Following the WABSA instructions [19], walkability and bikeability scores were classified into 5 categories: very poor, poor, fair, good and very good. The percentage of good and very good road segments for walkability-bikeability per school were calculated and used in the analysis.

Average monthly temperature for each school location was obtained from www.almanac.com/weather. If the exact location of the school was not available on the website, the closest neighboring city with information was identified. Temperature data were compiled for each of the 8months when data about walking and biking were collected.

#### School-level descriptive variables

Data about student enrollment, race/ethnicity, and number of students receiving free or reduced price lunch were collected for each school either through the initial WTS survey or from information publically available through various school or state websites.

### Statistical analysis

Total number of trips to and from school (including both active and passive transport modes) was calculated in order to check for errors and excessive absences from school on a given week. Travel recalls were excluded if the child reported more than 12 trips to and from school (indicating more trips than possible during the week) or less than 8 trips (indicating more than 1 day of missed school or non-reporting), thus reducing the number of usable recalls from 12,719 to 10,809 (85%).

Because participants’ data (parents and children, fall and spring) were not linked, all variables were summarized at the school level including: active school travel, parents’ and children’ perceived barriers and active school travel norms, distance to school, and walkability-bikeability. For those measured in the fall and spring (all variables except the active school travel), we examined if there were significant differences between the two time points. Because there were no significant differences, an average score was calculated and used in the subsequent analysis.

Generalized linear mixed models (GLMM) with log-link functions were used to assess bivariate and multivariate associations between individual variables and active school travel trips per week. The distribution of the main outcome (active school travel per week) was assumed to be Poisson because it was skewed to the left with many zero active school travel trips even though it was truncated at 10 travel trips to the right. Because student level active school travel data were clustered by school and time, each GLMM included time within school cluster as a random effect, and individual and school level variables as fixed effects. The intraclass correlation (ICC) for the number of times walking to and from school/week was calculated by adopting the approach described by Nakagawa and Schielzeth [20] for a generalized Poisson distribution. The ICC was 0.03 (95% C.I. 0.013-0.049) with a standard error of 0.01. All models were adjusted for distance by including the proportion of families living within 1 mile of school as a covariate because the distance was a strong predictor of active school travel and it changed estimates of relations (betas) drastically in many cases between each of other covariates and active school travel.

To build our final model, we started with a base model that included all variables with a substantial relationship (p < 0.15) with active school travel trips from the bivariate analyses, including distance, percentage of African American and Hispanic students, parent barrier factors (safety and weather, suitability of the route to school, child resistance), child barrier index, parent and child perceived social norms, walk/bikeability, and temperature. The parent barrier factor “no walking companion” was a significant in the bivariate analysis, but was not included in building the final model due to its strong correlation with another parent barrier factor “safety and weather” (r = 0.821). Based on our knowledge of the scientific literature, consensus among the authors and to minimize issues with colinearity only “safety and weather” was retained in the base model. This base model was reduced using a manual stepwise backward elimination approach in developing the final model. At each step, one variable was removed based on the significance of the parameter estimates (largest p-value) and the change in the −2 log-likelihood (smallest change when variable removed). The stepwise reduction continued until p-values for all parameters were < 0.15 and/or the change in the −2 log-likelihood was > 2 (df = 1) for any variable removed. This process resulted in removing four variables: percentage of African American students, children’s perceived barrier index, parents’ perceived active school travel norms, and children’s active school travel norms. All calculations were performed using the software packages of SAS version 9.2 and SPSS v.16.0 for Windows.

## Results

### Characteristics of the Schools

Characteristics of the schools including state location, level of WTS program implementation, student enrollment, race/ethnicity of students, rates of participation in the lunch program, frequency of active school travel, percentage of students within a walkable distance from school (1 mile) are shown in Table 1. Schools were located in nine states: Florida, North Carolina, Texas, Colorado, California, Alaska, Minnesota, Pennsylvania and New Jersey. The student enrollment, racial and ethnic distribution, and participation in the lunch program varied substantially among schools. The average monthly response rate to travel recall surveys among the 4^th^ and 5^th^ grade students from all the schools was 55.6%. However, response rates across schools and months ranged from 19.8% to 85.2%. On average, students engaged in 3.1 active school trips per week (out of 10 possible); and 35.5% were classified as active travelers (those reporting ≥4 active trips per week). Most students (58.2%) lived within 1 mile of their school. The average temperature from October to May across all schools was 51.7 Fahrenheit, but varied from a low of 25.1 (in Alaska) to a high of 69.1 (in Florida). Based on WABSA observations, 50% of the identified roads in 10 of 18 schools were in good or very good condition for walking and biking (data not shown).

**Table 1:** Descriptive characteristics of socio-demographic factors, active school travel and distance, stratified by schools

**Region & level**	**State**	**# Students**	**Race/Ethnicity (%)**				**Free/reduced lunch (%)**	Sample size^*^			**Active school travel/week**			**Distance**
			**White**	**AA**^**†**^	**Hispanic**	**Other**		**Response rate (%)**	**Surveys collected**	**# Months**	**Median (25**^**th**^**, 75**^**th**^**)**	**Mean (SD)**	**%**	**(% w/in 1 mile)**
CA-0	CA	606	6.4	2.0	88.6	3.0	80.4	51-72	587	8	5 (0,10)	5.0 (4.2)	59.5	72.5
CA-1	CA	321	24.6	3.1	70.7	1.2	75.1	31-48	283	5	1 (0,10)	3.7 (4.3)	41.7	60.2
CA-2	CA	547	43.3	27.1	19.6	8.8	88.5	65-72	506	6	5 (0,10)	4.8 (4.3)	54.9	92.0
CA-3	CA	479	74.9	0.4	19.2	5.0	11.7	42-64	695	8	0 (0,6)	2.8 (3.9)	30.1	39.7
NE-0	PA	342	31.3	23.1	41.2	4.4	71.9	55-76	325	5	10 (5,10)	7.1 (3.9)	77.8	96.3
NE-2	PA	402	92.8	4.5	1.2	1.5	16.7	72-100	917	8	1 (0,5)	2.9 (3.7)	36.3	78
NE-3	NJ	531	94.2	0.6	0.8	4.5	0.0	43-56	697	7	5 (0,9)	4.7 (4.1)	54.8	91.9
NW-0	AK	461	59.4	0.9	3.0	36.7	28.0	33-45	502	6	0 (0,1)	1.7 (3.2)	19.5	39.9
NW-1	AK	340	54.1	0.3	3.5	40.9	28.3	19-59	359	8	1 (0,6)	3.3 (4.0)	40.9	72.9
NW-2	MT	473	90.5	0.4	2.1	7.0	29.0	51-91	591	7	1 (0,8)	3.5 (4.1)	39.6	51.5
NW-3	AK	583	16.8	14.2	9.1	59.9	75.5	15-56	547	8	0 (0,2)	2.2 (3.8)	23.9	72.2
SC-0	CO	374	60.7	3.2	19.5	16.6	52.7	13-29	201	6	1 (0,8)	3.4 (4.1)	39.3	58.8
SC-1	TX	573	44.7	38.6	15.2	1.6	42.8	38-71	619	8	5 (0,8)	4.5 (3.9)	58.2	74.7
SC-2	CO	427	81.0	3.3	11.7	4.0	22.7	13-57	249	8	0 (0,6)	2.9 (4.1)	34.1	65.9
SC-3	TX	347	67.9	8.6	18.4	5.1	9.4	70-84	571	8	0 (0,2)	1.8 (3.3)	20	40.9
SE-3	FL	850	45.8	53.0	0.6	0.6	69.9	35-72	433	8	0 (0,0)	1.2 (2.9)	12.5	47.6
SE-0	FL	371	51.9	32.4	12.7	3.1	52.7	69-94	1642	8	0 (0,0)	1.3 (3.1)	15.2	24.3
SE-2	NC	349	73.3	11.9	10.0	4.9	55.3	74-91	1085	8	0 (0,10)	3.1 (4.4)	34	50.2

*Sample size for the main outcome (active school travel). The response rate is expressed with the range along the different measures (lowest –highest), surveys collected for each school (N total = 10,809 surveys) and number of the monthly measures (maximum 8 -from October until May). ^†^Africa American.

### Correlates of active school travel

The bivariate analysis (Table 2) found that the following factors were significantly related to children’s active school travel: percentage of students living within a 1-mile radius (Estimate = 0.0276, p < 0.0001), percentage of students who are African American (Estimate = −0.0099, p = 0.0002) or Hispanic (Estimate = 0.0075, p < 0.0001), walk/bikeability (Estimate = 0.0034, p = 0.0283), four of the parent’s perceived barrier factors (safety and weather (Estimate = −0.0272, p < 0.0001), suitability of the route (Estimate = −0.0175, p = 0.0030), no walking companion (Estimate = −0.0308, p < 0.0001) and children’s resistance (Estimate = −0.0627, p < 0.0001)), children’s perceived barriers (Estimate = −0.0431, p < 0.0001), and perceived active school travel norms by parents (Estimate = 0.0166, p < 0.0001) and children (Estimate = 0.0158, p = 0.0002). Higher percentage of Hispanic students, walk/bikeability scores, and parents’ and children’s perceived active school travel norms were associated with higher levels of active school travel. However, higher percentage of African American students and parents’ perceived barriers were associated with lower levels of active school travel.

**Table 2:** Descriptive characteristics of the total sample and independent bivariate analysis with active school travel per week

		Bivariate associations with active school travel per week^*^		
	**Mean ± SD**	**Estimate**	**t value**	***p***
Distance (*% within 1 mile*)	62.76 ± 20.33	0.0276	13.62	**<0.0001**
Socio-demographic factors (% *of students*)				
White	56.31 ± 25.97	0.0011	0.70	0.4856
African American	12.64 ± 15.74	−0.0099	−3.83	**0.0002**
Hispanic	19.28 ± 24.33	0.0075	4.80	**<0.0001**
Free or reduced lunch	45.03 ± 27.74	−0.0021	−1.36	0.1748
Physical factors				
Temperature (°*F*)	50.50 ± 15.43	0.0047	1.74	0.0851
Walk/bikeability (% *good or very good*)	48.92 ± 26.68	0.0034	2.22	**0.0283**
Parents’ perceived barriers (% *of barriers endorsed by parents*)				
Safety and weather	34.38 ± 8.33	−0.0272	−6.03	**<0.0001**
Suitability of the route to school	19.73 ± 7.14	−0.0175	−3.03	**0.0030**
Time issue	15.85 ± 4.90	−0.0009	−0.11	0.9148
No walking companion	18.84 ± 5.94	−0.0308	−4.75	**<0.0001**
Children’s resistance	7.67 ± 4.38	−0.0627	−7.94	**<0.0001**
Parents’ perceived active school travel norms (% *perceiving others walking*/*biking to school with children every day and a few times a week*)	53.61 ± 15.04	0.0166	4.09	**<0.0001**
Children’s perceived barriers (% *of barriers endorsed by children*)	35.67 ± 4.72	−0.0431	−5.81	**<0.0001**
Children’s perceived active school travel norms (% *perceiving other kids walking*/*biking to school*)	56.32 ± 12.81	0.0158	3.85	**0.0002**

* Bivariate associations were adjusted for distance and clustering (school*time). Distance adjusted for clustering only.

The stepwise reduction of the base model resulted in removing four variables: percentage of African American students, children’s perceived barriers, parents’ perceived active school travel norms, and children’s active school travel norms (Table 3). The final model included distance (Estimate = 0.0301, p < 0.0001), percentage of Hispanic students (β = 0.0059, p < 0.0001), temperature (Estimate = 0.0034, p = 0.1285), walk/bikeability (β = 0.0031, p = 0.0255), safety and weather (Estimate = −0.0245, p < 0.0001), suitability of the route (Estimate = 0.0182, p < 0.0018), and children’s resistance (Estimate = −0.0438, p < 0.0001). Temperature was the only variable in the final model with a p-value >0.05. Parents’ perceived safety and weather and children’s resistance were found to have negative association with active school travel while all other factors (i.e., percentage of Hispanic students, walk/bikeability scores and parent’s perceived suitability of the route) had a positive association with active school travel.

**Table 3:** Estimates for assessing associations between active school travel and each of covariate in the base and final models

	**Base model**			**Final model**		
	**Estimate**	**t-value**	***p***	**Estimate**	**t-value**	***p***
Intercept	−0.0989	−0.17	0.8686	−0.4832	−2.13	0.0353
Distance	0.0275	6.00	**< 0.0001**	0.0301	16.60	**<0.0001**
White	*Not in b/c bivariate p > 0.15*					
African American	−0.0006	−0.15	0.8795			
Hispanic	0.0052	2.58	**0.0110**	0.0059	4.23	**<0.0001**
Temperature (*°F*)	0.0030	1.17	0.2429	0.0034	1.53	0.1285
Walk/bike-ability	0.0024	1.38	0.1695	0.0031	2.26	**0.0255**
Safety and weather ^*^	−0.0160	−1.53	0.1280	−0.0245	−4.27	**<0.0001**
Suitability of the route to school	0.0102	1.07	0.2868	0.0182	3.19	**0.0018**
No walking companion	*Not in b/c covary with Safety and Weather (r = 0.821)*					
Children’s resistance	−0.0453	−4.22	**<0.0001**	−0.0438	−4.83	**<0.0001**
Parents’ perceived active school travel norms	−0.0005	−0.09	0.9292			
Children’s perceived barriers	−0.0096	−0.85	0.3975			
Children’s perceived active school travel norms	0.0018	0.36	0.7184			

All models were adjusted for clustering (school*time) and distance (% within 1 mile of school).

Base model was reduced in a stepwise fashion in building final model. One variable was removed at each step based on the significance of the parameter estimates (largest p-value greater than 0.15) and the change in the log-likelihood (smallest change when variable removed). The stepwise reduction continued until p-values for all parameters were < 0.15 and/or change in log-likelihood > 2.00 (df = 1) for any variable removed. This process resulted in removing 4 variables: percentage of African American students, children’s perceived barriers, parents’ perceived active school travel norms, and children’s active school travel norms.

*The variable “safety and weather” was strongly correlated with “no walking companion” (r = 0.821); for this reason only one was retained in the base model.

## Discussion

This study found that active school travel was mainly related to a combination of parent’s perceptions, socio-demographic and environmental variables, even after adjusting for distance to school. More specifically, we observed that higher rates of active school travel were found in schools where parents perceived low levels of children’s resistance to active school travel, were less concerned about safety and inclement weather and, unexpectedly, perceived the route as having more environmental challenges to navigate (suitability). We also found higher rates of active travel at schools with a higher percentage of Hispanic students and where roads and pathways to school were more pedestrian and cyclist friendly. The three more significant correlates for active travel (p < 0.0001) from the model were parents’ perception of children’s resistance, parents’ perception of safety and weather and the percentage of students who were Hispanic.

Parents’ perceptions of children’s resistance seem to play a key role on the decision of walking or cycling to school in the current study. Similar results have been reported in 5–6 year old [21] and 9–11 year old [22,23] Australian children. In the first study with younger children, parents’ perception of their child’s lack of “energy” was correlated to active school travel. In the studies with older children, the parents’ perceptions that their child doesn’t like to walk was negatively associated with active school travel while perceptions that the child preferred to walk was positively associated. The child’s own perceptions and decisions have also been reported to be essential in the decision-making process leading to his/her engagement in active school travel [16]. Although items about child resistance were included in both parent and child surveys’ questions about barriers, it only emerged as a separate factor in the parent survey. And because the parent and child survey data were not linked, it is difficult to distinguish whether it is actual child resistance or parent perceptions of child resistance that is having the greatest impact on active school travel.

While our exploratory factor analysis of parents’ perceived barriers combined issues of safety and weather, with the final model showing them to be inversely associated with active school travel, the literature has generally looked at these issues separately. An association with safety has been regularly evidenced in the literature with parents reporting concerns about traffic safety (e.g., dangerous street crossings), poor pedestrian access to school (e.g., missing or incomplete sidewalks), and crime threats (e.g., bullies) [2,10-12,24]. A qualitative study focused in the parents’ decision making process of their children’s school travel mode indicated that the primary decisions were related to safety issues (e.g., traffic, strangers) [25]. However other studies have shown those children’s [26,27] and adolescent’s [28] perceptions of safety are generally unrelated to whether they walked to school. Hence, safety perceptions from parents and children seem to diverge. These divergent perceptions were illustrated in a study by Olvera et al., which showed that children perceived their neighborhood safer than their mothers [29]. Lorenc et al. reported that children and young people would like to walk and cycle more and be more independently mobile, but were restricted by their own and their parents’ concerns about safety [10].

Similarly, the literature shows an association between active school travel and weather. Lorenc et al.’s literature review of active school travel correlates identified three different studies assessing the influence of weather [10]. They reported that bad weather was widely regarded as a disincentive to walking or cycling. Similarly, two longitudinal studies have shown seasonal variability, especially for cycling, supporting the influence of weather on patterns of commuting to school among youth from Norway [30] and US [31]. However, findings have not always been consistent; others in the US and Australia have found that parents’ perceptions of weather and objective weather assessment related very little to active school travel [32]. Understanding that parents and children are hesitant to walk in rainy or very cold weather should be used with knowledge about local seasonal weather patterns to inform practitioners and interventionist about the best time of year to promote active travel.

The final model also indicated that schools with a higher percentage of Hispanic students had a higher percentage of active travelers, but percentage of African American students was not a significant predictor. Findings are difficult to compare to previous studies as the literature has shown mixed results. A 2008 review concluded that minorities (Hispanics and African Americans) were more likely to engage in active school travel [2]. However, studies published since that review have often found no significant association between active school travel and Latino ethnicity [9,33-37] or African American race [10,34-37]. Other studies have even shown ethnicity and race to be associated with lower rates of active school travel [9,37,38]. The relationship between ethnicity and active travel seen in the current and earlier studies is hypothesized to reflect cultural norms in which Hispanic adults are more likely to walk for transportation and leisure [39]. However, Mendoza et al. observed that level of acculturation was inversely associated with active school travel among Latino children [33]. Hence the lack of association seen in more recent studies may reflect greater acculturation of those samples. In the present study, level of acculturation was not assessed, so it is impossible to understand to what degree that may explain the association observed. McDonald et al. [35] were able to determine that a major factor in these relationships between ethnicity and race with active school travel were due to distance between home and school, with a greater percentage of Hispanic (35%) and African American (22%) children living within 1 mile of school compared to white (16%) children. This may help explain why in our final model, which controlled for distance, race was no longer a significant predictor of active school travel. The negative association observed in the bivariate analysis may have been a reflection of African American children living a farther distance from school. Another underlying factor identified by McDonald et al. influencing this relationship between ethnicity/race and active school travel was income [35]. Income is commonly studied socio-demographic variable, and generally, studies indicate that children from a low socio-economic status (SES) background are more likely to walk or bike to school than children with a higher SES [3,34,37,40,41]. In the present study, household income was not assessed, but the percentage of students receiving free or reduced lunch (an indicator of SES at the school level) was not significantly related to active school travel. While this school-level variable is not a perfect assessment of income, Su et al. were able to demonstrate a positive association with active school travel using a very similar school-level indicator [37]. This interplay of ethnicity, race, acculturation, and income is a complex issue that warrants additional exploration in future research.

Overall the findings suggest that future active school travel intervention efforts should incorporate strategies to work with parents in order to identify the safest route to school or create monitored routes to reduce anxiety about safety issues, improve parent–child communications about active transportation which may help to clarify misperceptions around child’s interest, and to encourage and support active school travel during the most appropriate time of the year. Because distance is so strongly related to active school travel it must be considered when interventions are planned. This could be as simple as targeting only families within a certain distance, or distance to school could be incorporated into a more complex intervention model to help determine the intensity of the intervention for a given family. One model that addresses many of these issues and has been used effectively in a number of communities is the Walking School Bus (WSB) [42]. WSB is a walk-to-school program where children walk to school in groups along a set route (and with set stops along with way), with adults (e.g., parents) essentially serving as the bus driver for supervision. It may be a good strategy to: encourage children to walk together, get parents involved in their children’s active school travel, and be a visible sign of active school travel that models this behavior for the whole community [42].

### Study limitations and strengths

The main limitation of the current study was that child and parent data were not linked and individual child participation was not followed over time. Therefore, all data had to be analyzed at the school-level. Child travel data, which were collected monthly but not matched over time, limit our ability to describe the change in active school travel for individual children. Finally, these data were part of a national evaluation study that occurred over 10 years ago. While there have been national policy initiatives like Safe Routes to School implemented in the interim, funding for such programs has been limited and is estimated to have reached only 10% of schools [9]. The stability in rates of active school travel would suggest that determinants of this behavior have not changed drastically. Therefore, the information presented is believed to remain very relevant for today despite the time lapse between the evaluation and the current analysis.

In spite of these limitations, this study had several strengths. The main strength was the large number of surveys (n = 10,809) that were collected from children over eight consecutive months and the amount of survey information gathered from hundreds of children and their parents from 18 different schools across the US. This information provides a unique national perspective for active school travel patterns. We also used a continuous variable as the main outcome (i.e., active travels per week) ranging from 0 to 10 weekly travels. Most studies include a dichotomized variable of active school travel (active vs. non-active) which may limit their ability to detect the effect certain variables on active school travel, especially given the large number of children that use more than one mode of travel. Finally, we adjusted for distance in all the analysis allowing us to identify factors that relate to active school travel independent of distance to school.

## Conclusions

In conclusion, controlling for the fact that distance between home and school is an important predictor of active school travel, parents’ perceptions of their children’s resistance to active school travel and parental concerns about safety and weather travel to school are the primary modifiable correlates for active school travel among 4^th^ and 5^th^ grade children in the US. Future interventions to promote active school travel should more actively engage parents and address these concerns. Programs like the Walk to School program, which are organized by the schools and can engage community resources like public safety officials, could help overcome many of these perceived barriers to active transport.

## Competing interests

The authors declare that they have no competing interests.

## Authors’ contributions

DW designed the original study and served as the principal investigator providing scientific oversight for all activities. AV served as the project manager and oversaw the data collection and data cleaning. PC, DH, ZG and AN analyzed and interpreted the data. PC led the development the manuscript, with contributions from all authors. All authors reviewed and approved the final manuscript.

## Supplementary Material

Additional file 1 Exploratory factor analysis: Parent’s barriers. Click here for file
