# Supplementary material for: A cross-sectional study of demographic, environmental and parental barriers to active school travel among children in the United States
Source: Int J Behav Nutr Phys Act. 2014 May 9;11:61. doi: 10.1186/1479-5868-11-61 (PMC4032634; doi:10.1186/1479-5868-11-61)
Supplement: Additional file 1 — Exploratory factor analysis: Parent’s barriers. [file 1479-5868-11-61-S1.docx]

Exploratory Factor Analysis: Parent´s barriers

- Parents were asked to identify reasons why they:
  - “cannot or do not allow their child to walk or bike to school”
  - a checklist of 22 barriers commonly reported in the literature and identified from formative work
- Using exploratory factor analysis we identified:
  - five barrier factors using 20 of the original 22 items (see below in Table 1)
  - each barrier item was scored 0 (no) or 1 (yes)
  - an average score was computed for each barrier factor.

Table S1. Exploratory factor analysis of the parent´s barriers of active school travel. Values are rotated factor loadings.

| **Barrier factors:**  **Items:** | **External safety and weather**  **(6 items)** | **Suitability of the route**  **(6 items)** | **Time issues**  **(3 items)** | **No walking companion**  **(3 items)** | **Child resistance**  **(2 items)** |
| --- | --- | --- | --- | --- | --- |
| Bullies | .819 |  |  |  |  |
| Kidnapping | .782 |  |  |  |  |
| Arriving safely to school | .621 |  |  |  |  |
| Weather | .508 |  |  |  |  |
| Traffic | .432 |  |  |  |  |
| Unleashed dogs | .413 |  |  |  |  |
|  |  |  |  |  |  |
| Lack of sidewalks |  | .785 |  |  |  |
| Lack of crosswalks |  | .622 |  |  |  |
| Step hills |  | .605 |  |  |  |
| Areas without people around |  | .507 |  |  |  |
| Speed and traffic |  | .455 |  |  |  |
| Insufficient daylight in morning |  | .431 |  |  |  |
|  |  |  |  |  |  |
| Lack of time in the morning |  |  | .752 |  |  |
| Lack of time in the afternoon |  |  | .690 |  |  |
| More convenient to drop-off/pick-up |  |  | .481 |  |  |
|  |  |  |  |  |  |
| No other kids to walk with |  |  |  | .821 |  |
| No other adults to walk with |  |  |  | .690 |  |
| Conflicts with work schedule |  |  |  | .551 |  |
|  |  |  |  |  |  |
| Child too tired |  |  |  |  | .792 |
| Child does not want to walk or bike |  |  |  |  | .745 |
|  |  |  |  |  |  |
